# Mo-Doped Ni/C Catalyst for Improved Simultaneous Production of Hydrogen and Carbon Nanotubes through Ethanol Decomposition

**DOI:** 10.3390/nano14141205

**Published:** 2024-07-16

**Authors:** Jinxiang Diao, Xiaojie Liu, Xianmeng Wang, Yuzhu Zhang, Jingkai Yue, Hui Wang

**Affiliations:** 1 School of Aeronautical Maintenance Engineering, Aeronautical Polytechnic Institute, Xi’an 710089, China; wxm226226@163.com (X.W.); zhangyuzhu1110@163.com (Y.Z.); kw1013@163.com (J.Y.); 2 Key Laboratory of Synthetic and Natural Functional Molecule Chemistry (Ministry of Education), College of Chemistry & Materials Science, Northwest University, Xi’an 710069, China; xiaojie.liu@nwu.edu.cn (X.L.); huiwang@126.com (H.W.)

**Keywords:** multi-wall carbon nanotubes, hydrogen production, Mo-Ni/C catalyst, ethanol decomposition

## Abstract

A Mo-Ni/C catalyst was developed and assessed in terms of the decomposition of ethanol to produce multi-wall carbon nanotubes (MWCNTs) and hydrogen. The catalyst utilized different molar ratios of Mo:Ni (1:9, 2:8, and 3:7), with Mo acting as a dopant to enhance the MWCNT yield and Ni acting as the primary active phase for MWCNT formation. Among the tested ratios, the 2:8 Mo:Ni ratio exhibited the optimal performance, yielding 86% hydrogen and high-quality MWCNTs. In addition to hydrogen, the process also generated CO, CH_4_, and CO_2_. Gas chromatography (GC) was employed to analyze the influence of the Mo:Ni ratio on gas production and selectivity, while the quality of the resulting MWCNTs was evaluated using SEM, Raman spectroscopy, and TEM analyses.

## 1. Introduction

Hydrogen is increasingly recognized as a clean energy alternative to traditional fossil fuels due to its minimal environmental impact [1,2]. Various methods of hydrogen production have been explored, such as water electrolysis [3], water photolysis [4], and the partial oxidation and reforming of hydrocarbons [5,6]. Among these methods, the thermochemical decomposition of ethanol and catalytic cracking of ethanol are gaining prominence as viable approaches to hydrogen generation [7,8]. Through these processes, ethanol can be effectively transformed into hydrogen and CNTs using heterogeneous catalysts [9,10]. The challenge of catalyst deactivation due to carbon deposition, including fouling, coking, and filament formation, remains a significant issue in hydrogen production from ethanol [11,12]. Research on the mechanism of hydrogen production from ethanol is limited [13,14], while CNTs are recognized as valuable carbon nanomaterials with unique properties and diverse applications [15,16]. To achieve high-quality CNTs and pure hydrogen, the development of nano-catalysts is crucial for the efficient decomposition of ethanol [16,17]. Transition elements like Fe, Co, Ni, and Mo are commonly utilized as active catalysts of CNT growth [18,19,20]. Nickel-based catalysts, in particular, have been extensively studied for their high activity at lower reaction temperatures, though they may undergo quicker deactivation at temperatures exceeding 500 °C [21,22,23].

Sophisticated techniques and advancements are required to achieve uniform CNT growth on nickel particles. Extensive efforts have been devoted to exploring the enhancement of both MWCNTs’ productivity and their quality through the introduction of a secondary metal [24]. The incorporation of molybdenum (Mo) into cobalt catalysts has been shown to significantly increase the yield and improve the quality of MWCNTs [25]. Additionally, studies have demonstrated that an addition of Mo can enhance the catalytic performance of Fe/CeZrO_2_ catalysts during hydrogen production, as reported by Ramasubramanian and colleagues [26].

In this study, ethanol is employed for the concurrent synthesis of CNTs and hydrogen through chemical vapor deposition utilizing Ni/C and Mo-Ni/C catalysts. A set of Ni/C and Mo-Ni/C catalysts are synthesized and assessed for their effectiveness in catalyzing ethanol’s decomposition. The impact of a Mo incorporation into the Ni/C catalyst on the generation of CNTs and hydrogen is thoroughly examined. The optimal reaction parameters for achieving high-quality MWCNTs and maximizing the hydrogen yield from ethanol decomposition are identified and discussed.

## 2. Experimental Section

### 2.1. Catalysts Preparation

A catalyst composed of Mo-Ni supported on graphite was synthesized utilizing a wetness impregnation method [27]. Initially, 3 g of graphite powder was immersed in an aqueous solution containing specified quantities of Ni(NO_3_)_2_·6H_2_O and (NH_4_)_6_Mo_7_O_24_·4H_2_O to create a uniform solution. The impregnated samples underwent evaporation drying at 90 °C to yield a dried catalyst powder. Subsequently, the dried samples were subjected to calcination at 300 °C for 5 h, followed by reduction with hydrogen at 773 K for 1 h. The prepared catalysts were denoted as Mo-Ni (Mo: Ni = X)/C; the total metal amount was 5 wt% ((Mo + Ni)/(Mo + Ni + C) = 5 wt%) in the supported catalyst and the molar ratio of Mo:Ni in the catalysts was adjusted to be 1:9, 2:8, or 3:7. A Ni/C catalyst was used as a contrast. The Ni loading in Ni/C was tailored to constitute 5 wt% of the total weight (Ni/(Ni + C) = 0.05) (Table S1).

### 2.2. Decomposition of Ethanol

**Figure 1:**
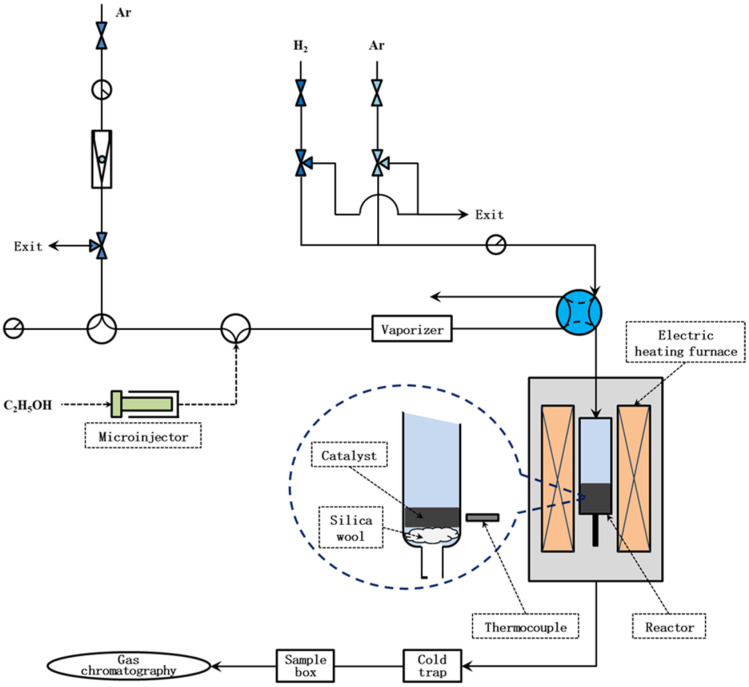
Schematic diagram of simultaneous production process used to convert ethanol into MWCNTs and hydrogen.

Hydrogen and CNTs were co-synthesized via the ethanol decomposition process over Mo-Ni/C and Ni/C catalysts. The experimental apparatus is schematically shown in Figure 1. The experiment was carried out on a standard vertical fixed bed centrally located within a quartz tubular reactor with an inner diameter of 5 mm. Before initiating ethanol decomposition, 150 mg of catalyst underwent hydrogen reduction at 500 °C for 1 h, with a gas flow rate of 40 mL/min comprising a 1:1 mixture of H_2_ and Ar, and was subsequently purged with Ar (40 mL/min) for 40 min. The ethanol decomposition reaction was carried out over the Mo-Ni/C catalyst within a temperature range of 500–700 °C and with a heating rate of 5 °C/min. Upon reaching a stable temperature, the ethanol was injected into a vaporizing chamber (operating at 100 °C), using a controllable injector, at a flow rate of 0.3 mL/h and then transported to the tubular reactor using Ar (40 mL/min) as the carrier gas. Throughout the reaction, the gas composition of the outlet stream was analyzed using gas chromatography (GC). After 60 min, ethanol feeding was ceased, and the solid products were collected from the tubular reactor.

### 2.3. Characterization

Samples were subjected to a Powder X-ray diffraction (XRD) analysis using Cu Kα radiation on a Bruker D8 ADVANCE diffractometer. The morphology and nanostructure of the CNTs were examined using a transmission electron microscope (TEM, G2 F20 S-TWIN), high-angle annular dark-field-scanning transmission electron microscopy (HAADF-STEM) instrument (Themis Z, FEI, USA) operating at 300 kV, and a scanning electron microscope (SEM, SU8220, Hitachi, Japan) at 15 kV. Raman scattering studies of the CNTs were conducted utilizing a Raman spectrometer (LabRAM HR800, HORIBA Jobin Yvon, France) with a laser wavelength of 514 nm.

### 2.4. Evaluation of Catalysts

The performance of Mo-Ni/C and Ni/C catalysts in the ethanol decomposition process was assessed based on their effectiveness in generating hydrogen, converting ethanol, and selectively producing different gas products; their CNT yield; and the CNTs’ purity [28]. These evaluations were conducted using Equations (1)–(5), respectively.

(1) $$H_{2}\;\mathrm{yield}\;(\%)=\frac{\mathrm{mole}\;\mathrm{of}\;H\;\mathrm{atom}\;\mathrm{converted}\;\mathrm{to}\;H_{2}}{\mathrm{theoretical}\;\mathrm{mole}\;\mathrm{of}\;H\;\mathrm{atom}\;\mathrm{contained}\;\mathrm{in}\;\mathrm{ethanol}\;\mathrm{feed}\;}\times 100$$

(2) $$\mathrm{Ethanol}\;\mathrm{conversion}\;(\%)=\frac{\mathrm{mol}\;(\mathrm{ethanol})\;\mathrm{in}-\mathrm{mol}\;(\mathrm{ethanol})\;\mathrm{out}\;}{\mathrm{mol}\;(\mathrm{ethanol})\;\mathrm{in}\;}\text{×}100$$

(3) $$S_{i}=\frac{n_{i}}{n_{\mathrm{total}}}\times 100$$

(4) $$\mathrm{CNTs}\;\mathrm{percent}\;\mathrm{yield}\left(\%\right)=\frac{m_{\mathrm{product}}-m_{\mathrm{catalyst}}}{m_{\mathrm{carbon},\mathrm{in}}}\times 100\%$$

(5) $$\mathrm{CNTs}\;\mathrm{purity}\;(\%)=\frac{m_{\mathrm{carbon}}-m_{\mathrm{carbon},\mathrm{support}}}{m_{\mathrm{product}}}\times 100\%$$

where n_i_ is the mole of i gas product (i = H_2_, CO, CH_4_, or CO_2_) and n_total_ is the total mole of all the gas products (H_2_ + CO + CH_4_ + CO_2_). m_product_ is the mass of the final product including the catalyst, m_catalyst_ is the mass of the reduced catalyst, and m_carbon,in_ is the mass of the total carbon in the feed. m_carbon_ is the mass of the total carbon product including support graphite.

## 3. Results and Discussion

### 3.1. Effect of Temperature and Amount of Mo Addition on Ethanol Decomposition

Ethanol is utilized as the primary feedstock in the chemical vapor deposition (CVD) process, which serves as the key catalytic step in reaction (6). Concurrently, CVD is employed to decompose CH_4_ into (CNTs) and hydrogen, leading to the generation of syngas (CO + H_2_) in reaction (7). The combined reactions (6) and (7) result in reaction (8), highlighting the thermodynamic preference for reaction (3) over reactions (1) and (2) at elevated temperatures [29].

(6) $${\mathrm{CH}}_{3}{\mathrm{CH}}_{2}\mathrm{OH}\to \mathrm{CO}+H_{2}+{\mathrm{CH}}_{4},\;\Delta H^{\vartheta }=49.76{\;\mathrm{kJ}\;\mathrm{mol}}^{-1}$$

(7) $${\mathrm{CH}}_{4}\to C+{2H}_{2},\;\Delta H^{\vartheta }=74.81{\;\mathrm{kJ}\;\mathrm{mol}}^{-1}$$

(8) $${\mathrm{CH}}_{3}{\mathrm{CH}}_{2}\mathrm{OH}\to C+\mathrm{CO}+3H_{2},\;\Delta H^{\vartheta }=124.58{\;\mathrm{kJ}\;\mathrm{mol}}^{-1}$$

**Figure 2:**
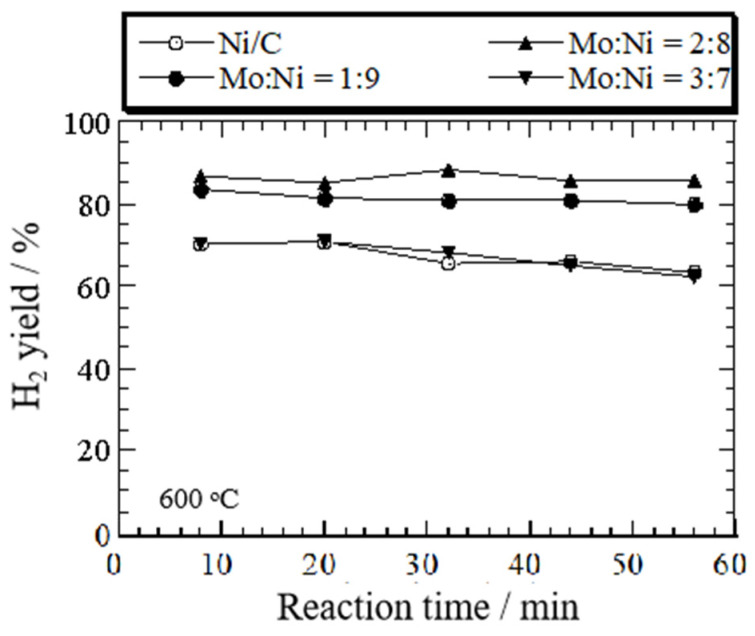
Change in H_2_ yield as a function of the reaction time during ethanol decomposition over Ni (5 wt%)/C and Mo-Ni/C catalysts with different molar ratios of Mo:Ni, 1:9, 2:8, and 3:7, at 600 °C.

Figure 2 illustrates the impact of the reaction temperature and the Mo:Ni molar ratio on the H_2_ yield during ethanol decomposition over Ni/C and Mo-Ni/C catalysts at 600 °C.

**Figure 3:**
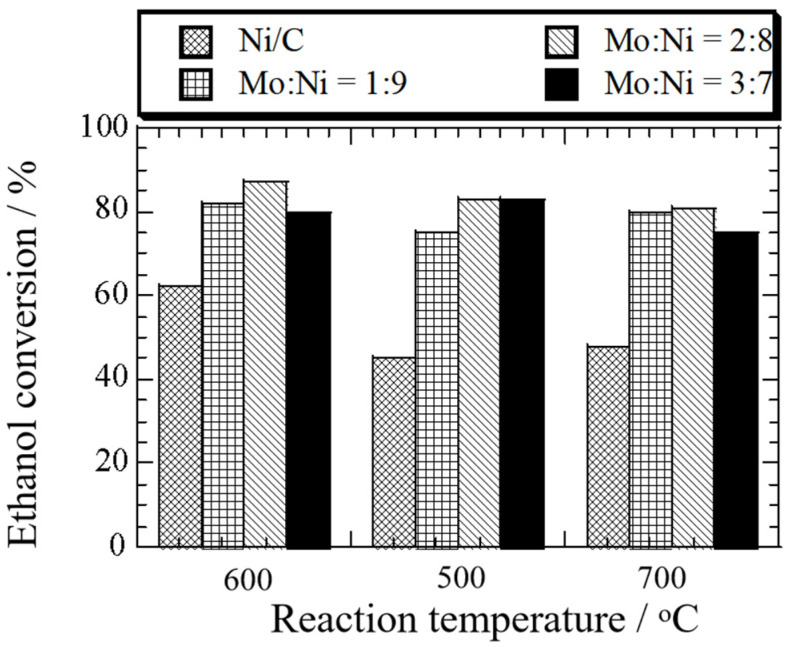
Change in H_2_ selectivity as a function of reaction temperature in ethanol decomposition over Ni (5 wt%)/C and Mo-Ni/C catalysts at different ratios of Mo:Ni—1:9, 2:8, and 3:7.

The figure reveals that the highest H_2_ yield is achieved at 600 °C for both the Mo-Ni/C and Ni/C catalysts, with the yield varying with temperature from 500 to 700 °C in the sequence 600 °C > 500 °C (Figure S1a) > 700 °C (Figure S1b). The most significant average H_2_ yield of 88% was recorded during ethanol decomposition over Mo:Ni(2:8) at 600 °C, with the yield changing with the Mo:Ni molar ratio at 600 °C in the sequence 2:8 > 1:9 > 3:7 > 0, at different ratios of 86%, 83%, 73%, and 70%. Additionally, Ni (5 wt%)/C exhibits deactivation, with a decrease in hydrogen yield over time. Consequently, it can be inferred that the incorporation of Mo into the Ni/C catalyst enhances the H_2_ yield and stabilizes the Ni/C catalyst at relatively higher temperatures (600 °C). The reason may be that the presence of NiMoOx improved the dispersion of Ni particles due to the Mo addition [30,31]. Figure 3 showed the H_2_ selectivity over the Mo-Ni/C on the basis of the reaction temperature.

**Figure 4:**
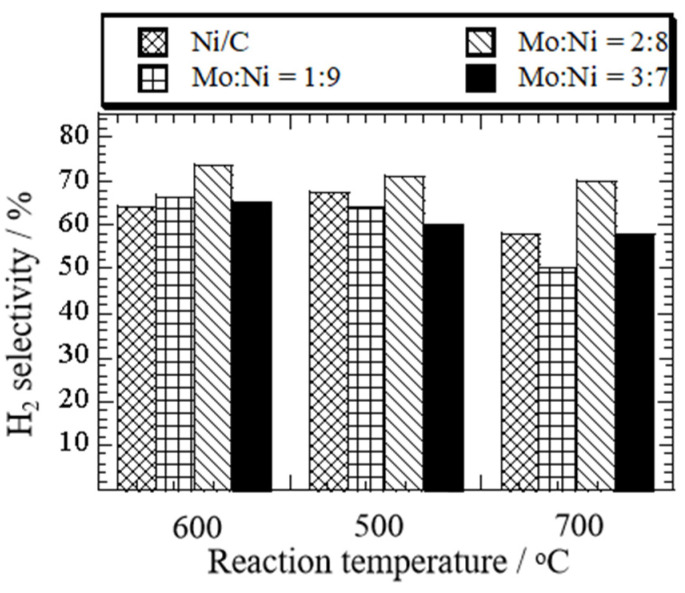
Effect of temperature and the ratio of Mo:Ni on ethanol conversion during ethanol decomposition over Ni (5 wt%)/C and Mo-Ni/C catalysts at different ratios of Mo:Ni—1:9, 2:8, and 3:7.

At 600 °C, hydrogen selectivity was the highest on the Mo-Ni (Mo:Co = 2:8)/C (72%), corresponding to the lowest CH_4_ selectivity (8%) (Figure S2a). The selectivity of CO (Figure S2b) and CO_2_ (Figure S2c) was 26% and 3%, respectively. In contrast, the selectivity of H_2_ was 64% over the Ni (5 wt%)/C. The results indicated that an appropriate Mo addition could improve hydrogen selectivity. When the molar ratio of Mo:Ni increased to 3:7, the hydrogen selectivity decreased. In Figure 4, the influence of reaction temperature and the molar ratio of Mo:Ni on ethanol conversion during ethanol decomposition over the Mo-Ni/C and Ni (5 wt%)/C catalysts is depicted.

As the ratio of Mo:Ni increased from 1:9 to 3:7, the rate of ethanol conversion increased firstly and then decreased at 500 °C, 600 °C, and 700 °C. When the ratio of Mo:Ni was 1:9 and the reaction temperature was 600 °C, the ethanol decomposition was highest (82%), which corresponded to the H_2_ yield and selectivity.

### 3.2. MWCNT Growth on the Mo-Ni/C Catalysts

**Figure 5:**
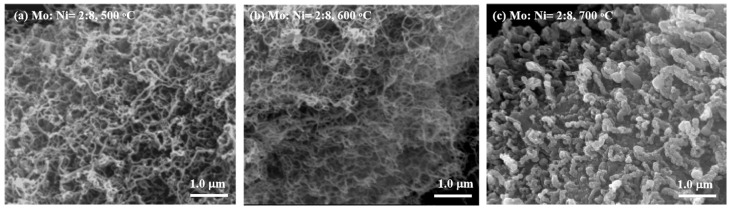
SEM images of MWCNTs formed by ethanol decomposition over the Mo-Ni (Mo:Ni = 1:9)/C catalyst at different temperatures: (**a**) 500 °C, (**b**) 600 °C, and (**c**) 700 °C.

SEM images of the MWCNTs produced via ethanol decomposition over the Mo-Ni/Mo:Ni = 2:8)/C catalyst are presented in Figure 5.

The images correspond to different synthesis temperatures: (a) 500 °C, (b) 600 °C, and (c) 700 °C, demonstrating the crucial role of temperature in CNT production. The SEM images indicate that MWCNTs were formed with metal particles at 500 °C. However, Figure 5b shows the length of CNTs with few metals and a little amorphous carbon formed over Mo:Ni (Mo:Ni = 2:8)/C. Figure 5c shows that carbon nanofibers (CNFs) with a wide diameter are formed at 700 °C, accompanied by the sintering of catalysts. These results indicated that the Mo-Ni (Mo:Ni = 2:8)/C catalyst at a temperature of 600 °C was more effective than that at temperatures of 500 °C or 700 °C in terms of its MWCNTs production from ethanol decomposition. The reason was that Mo is considered to have a role in dispersing metallic Ni at 600 °C. The Mo-Ni/C catalysts were sintered and reunited at relatively higher temperature of 700 °C, which led to larger Ni metal particles. The larger metal particles favored carbon fiber formation [32]. At 600 °C, with the ratio of Mo:Ni increasing from 0 to 3:7, the density of the CNTs declined over the Mo-Ni/C catalyst in Figure S3a–c. The presence of a high concentration of Mo led to increased crystallization of Ni, negatively impacting the quality of the CNTs. As a result, the Mo-Ni/C catalyst was found to be the most efficient for CNT production at 600 °C, with the optimal Mo:Ni molar ratio being 2:8. This could be attributed to the fact that, at 500 °C, the Ni (5 wt%)/C catalyst exhibited the highest number of active sites, possibly due to the prevention of Ni metal particle sintering at a relatively low temperature. One notable advantage of incorporating Mo into Ni/C catalysts is the ability to tailor the selectivity of the process towards MWCNT formation by adjusting the Mo-to-active-metal ratio [33,34,35].

**Figure 6:**
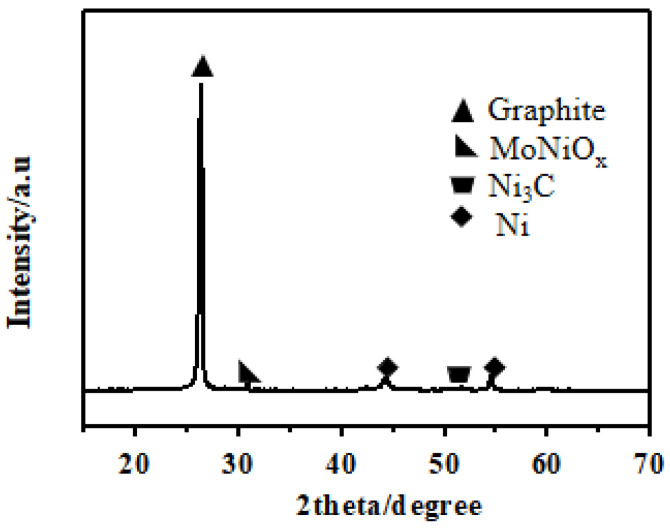
XRD pattern of MWCNTs on the Mo-Ni (Mo:Ni = 2:8)/C catalyst at 600 °C.

Figure 6 illustrates the XRD patterns of the CNTs synthesized using the Mo-Ni/C catalyst.

The presence of peaks at 2θ = 26° and 54.4° is indicative of diffraction peaks corresponding to the (002) and (004) planes of the graphitic tube walls of the CNTs [36] (JCPDS card no. #01-1235). In addition, the peak appearing at 2θ = 44° is attributed to Ni (JCPDS card no. #03-1051). The characteristic pattern of the MoNiO_4_ was 30.9° (PDF#16-0291). During ethanol decomposition, MoO_2_ (PDF#50-0739) (Figure S4) reacts with Ni to form MoNiO_4_, which prevented the extensive agglomeration of Ni species during the narrow window of CNT synthesis. Figure S5 displays the XRD patterns associated with the CNTs generated using the Ni (5 wt%)/C catalyst.

**Figure 7:**
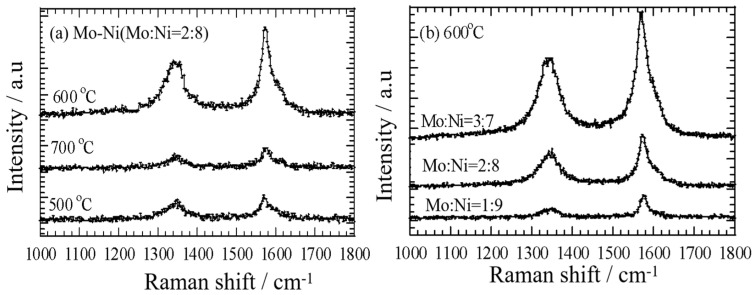
Raman spectra of MWCNTs on the Mo-Ni (Mo:Ni = 2:8)/C catalyst at temperature of 500 °C, 600 °C, and 700 °C (**a**,**b**) Mo-Ni/C catalysts with different ratios Mo:Ni (1:9, 2:8, and 3:7) at 600 °C.

Raman spectroscopy was utilized to evaluate the quality of the CNTs produced. In this analysis, the G-band observed at 1582 cm^−1^ signifies well-graphitized CNTs, while the D-band seen at 1335 cm^−1^ indicates the presence of disordered carbon, such as amorphous carbon or defects [37]. The I_G_/I_D_ intensity ratio, which reflects the ratio of graphitic to disordered carbon, serves as a metric for assessing the quality of the CNT products. A higher I_G_/I_D_ ratio is indicative of higher purity and lower defect levels in the nanotubes [38]. Figure 7a illustrates the Raman spectra of CNTs synthesized through ethanol decomposition using the Mo-Ni (Mo:Ni = 2:8)/C catalyst at different temperatures: 500 °C, 600 °C, and 700 °C.

It is evident that the I_G_/I_D_ ratio of the CNTs follows the temperature sequence of 600 °C (I_G_/I_D_ = 2.6) > 500 °C (I_G_/I_D_ = 1.5) > 700 °C (I_G_/I_D_ = 1.0). Additionally, the results indicate that the MWCNTs grown at 500 °C exhibited relatively higher purity and lower defect levels. In Figure 7b, the Raman spectra of the CNTs obtained from ethanol decomposition over Mo-Ni/C catalysts with varying Mo:Ni ratios (1:9, 2:8, and 3:7) at 600 °C are presented. The I_G_/I_D_ values of the CNTs produced at 600 °C are ranked as follows: Mo-Ni/C (Mo:Ni = 2:8) (I_G_/I_D_ = 2.6) > Mo-Ni/C (Mo:Ni = 1:9) (I_G_/I_D_ = 2) > Mo-Ni/C (Mo:Ni = 3:7) (I_G_/I_D_ = 1.7). The Mo-Ni (Mo: Co = 2:8)/C catalysts (30.5%) can produce higher CNT yields than Ni/C catalysts (21.8%) (Table S2). This confirms that additional Mo can greatly improve the CNT yield. These findings suggest that the CNTs synthesized over Mo-Ni/C (Mo:Ni = 2:8) at 600 °C exhibited good crystallinity in their graphite sheets, as well as lower levels of defects and impurities.

**Figure 8:**
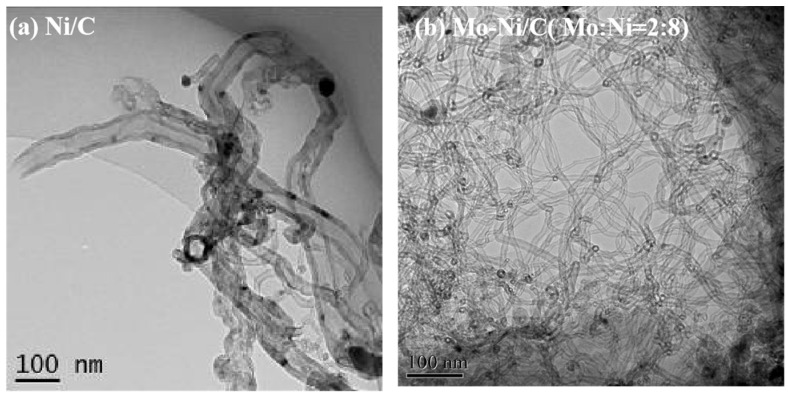
TEM images of MWCNTs on the Ni/C and Mo-Ni (Mo:Ni = 2:8)/C catalysts at a temperature of 600 °C.

The TEM images of the MWCNTs derived from ethanol decomposition over Ni/C and Mo-Ni (Mo:Ni = 2:8)/C at 600 °C are displayed in Figure 7. It is evident that the CNTs produced on both catalysts exhibit a multi-walled structure. Notably, the CNTs formed on the Ni/C catalyst (Figure 8a) display wider diameter distributions, albeit with a low yield. Despite this, the resulting samples exhibit high purity, with minimal impurities, long lengths, and clean walls (Figure 8b). An analysis of the HRTEM micrographs reveals the hollow structure of the CNTs. In particular, CNTs with a large outer diameter of 28 nm are observed on the Ni/C catalyst, likely attributed to nickel oxide agglomerations leading to the formation of large particles and, subsequently, large CNTs.

**Figure 9:**
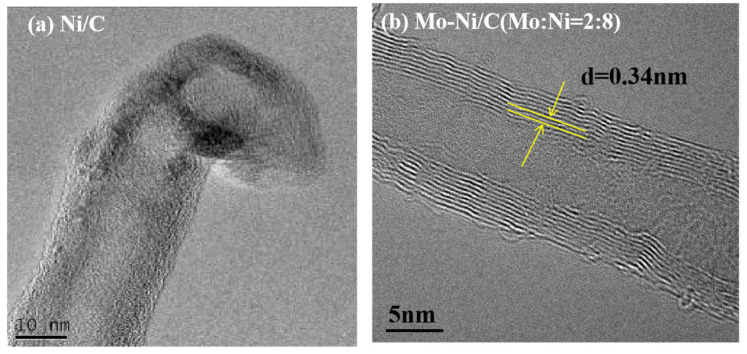
HRTEM images of MWCNTs on the Ni/C and Mo-Ni (Mo:Ni = 2:8)/C catalysts at a temperature of 600 °C.

Prior research has highlighted the relationship between metal catalyst particle size and nanotube diameter [39,40]. The graphite layers are not clearly discernible, and the catalyst particle is positioned at the tube’s apex (Figure 9a).

In contrast, CNTs formed on Mo-Ni (Mo:Ni = 2:8)/C exhibit an inner diameter ranging from 5 to 7 nm and an outer diameter between 12 and 15 nm. The tube walls are parallel to the tube axis, with a wall interval measuring approximately 0.34 nm (Figure 8b). Therefore, Mo-Ni (Mo:Ni = 2:8)/C exhibits excellent effectiveness comparable to analogous catalysts in recent works on producing H_2_ and MWCNTs simultaneously (Table S3).

## 4. Conclusions

This study delves into the impact of incorporating Mo into Ni supported on graphite for the synthesis of MWCNTs and hydrogen (H_2_) production within the temperature range of 500–700 °C. The Mo content within the catalyst exerts a significant influence on both the quality of the MWCNTs and the amount of hydrogen generated. Specifically, the Mo:Ni ratio of 2:8 demonstrates the optimal loading, showcasing the highest hydrogen yield (86%) and superior MWCNT quality, with increased yield and purity achieved at 600 °C. The resulting multi-walled CNTs exhibit an average outer diameter of 12–15 nm. The introduction of Mo serves to weaken the interaction between the Ni particles and the catalyst support. Additionally, the presence of Mo significantly suppresses the formation of amorphous carbon, thereby preserving the catalyst’s activity in facilitating the concurrent production of H_2_ and CNTs.

## Data Availability

Data are contained within the article and Supplementary Materials.

## Supplementary Materials

The following supporting information can be downloaded at https://www.mdpi.com/article/10.3390/nano14141205/s1, Table S1. The composition of the catalysts. Table S2. Summary of yields, purity, and I_G_/I_D_ ratio of CNTs synthesized over different catalysts at 600 °C. Table S3. The comparison of the H_2_ and CNTs yield of catalysts from the recent literature and this work. Figure S1. Change of H_2_ yield as a function of the reaction time. Figure S2. Change of CO, CH_4_ and CO_2_ selectivity as a function of reaction temperature. Figure S3. SEM images of MWCNTs formed by the ethanol over the Nib (5 wt%)/C and Mo-Ni/C catalysts. Figure S4 XRD pattern of Mo-Ni (Mo: Ni=2:8)/C catalyst. Figure S5. XRD pattern of MWCNTs over the Ni (5 wt%)/C catalyst.
