# Supplementary material for: Mo-Doped Ni/C Catalyst for Improved Simultaneous Production of Hydrogen and Carbon Nanotubes through Ethanol Decomposition
Source: Nanomaterials (Basel). 2024 Jul 16;14(14):1205. doi: 10.3390/nano14141205 (PMC11280427; doi:10.3390/nano14141205)
Supplement: Supplementary file 1 [file nanomaterials-14-01205-s001.zip › nanomaterials-3077018-supplementary.pdf]

Supplementary materials

Table S1 The composition of the catalysts used in this work and their parameters.

**Sample**	**(Mo+Ni)/(Mo+Ni+C) (wt%)**	**Mo:Ni (mole ratio)**
Mo:Ni (Mo:Ni = 1:9)/C	5	1:9
Mo:Ni (Mo:Ni = 2:8)/C	5	2:8
Mo:Ni (Mo:Ni = 3:7)/C	5	3:7
Ni/C	5	0

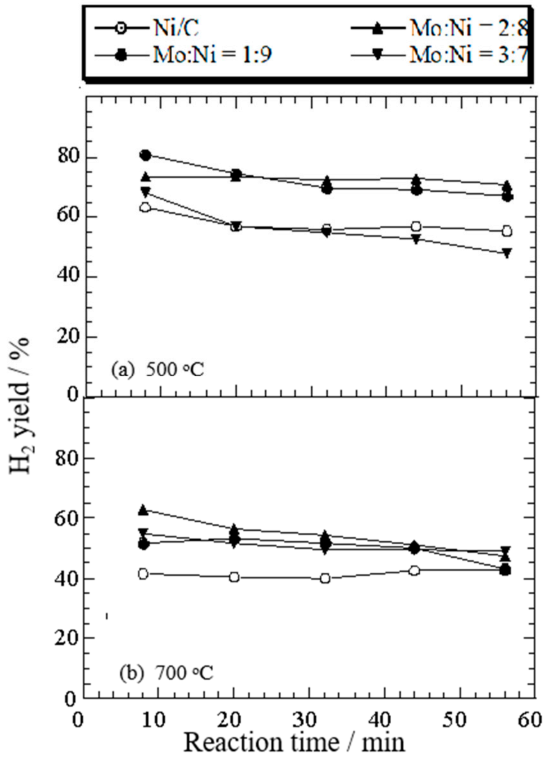

Figure S1. Change of H_2 yield as a function of the reaction time in ethanol decompostion over Ni(5wt%)/C and Mo-Ni/C catalyst with different moral ratio of Mo:Ni 1:9, 2:8 and 3:7 at 500 and 700 ºC.

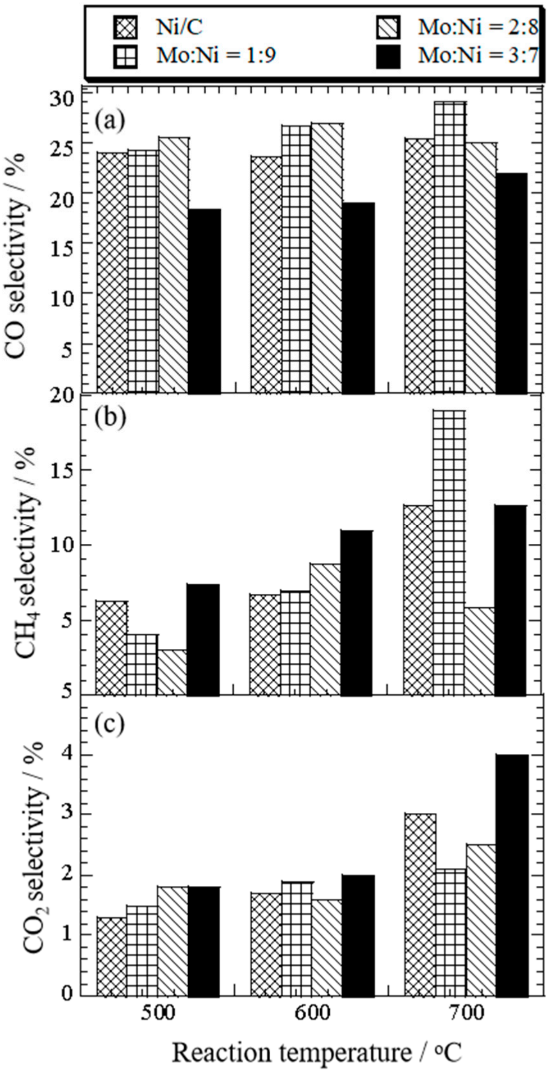

Figure S2. Change of CO, CH_4 and CO_2 selectivity as a function of reaction temperature in ethanol decompostion over Ni(5wt%)/C and Mo-Ni/C catalysts at different the ratio of Mo:Ni 1:9, 2:8 and 3:7.

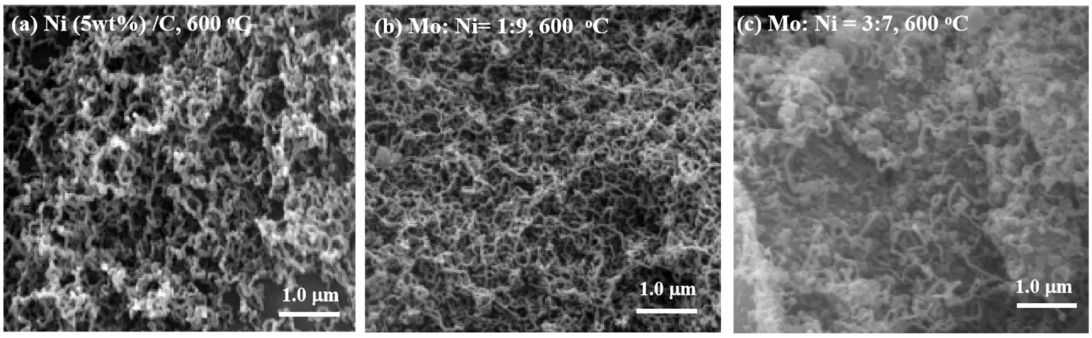

Figure S3. SEM images of MWCNTs formed by the ethanol over the Ni(5wt%)/C and Mo-Ni/C catalysts with different the ratio of Mo:Ni 1:9 and 3:7 at 600 ºC.

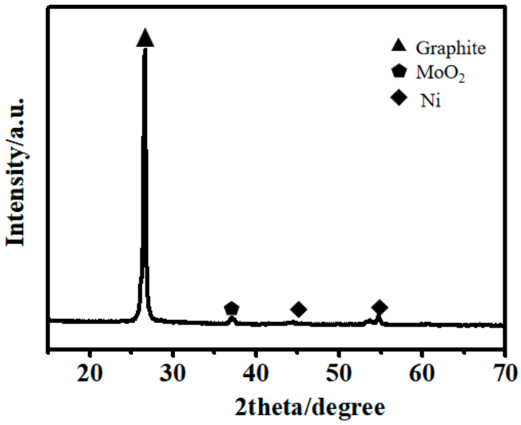

Figure S4 XRD pattern of Mo-Ni (Mo: Ni=2:8)/C catalyst.

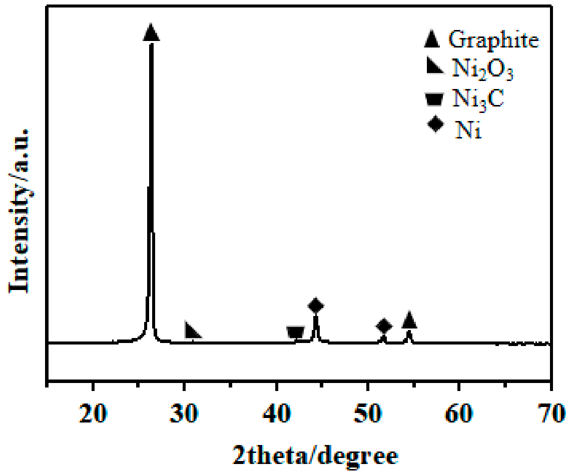

Figure S5. XRD pattern of MWCNTs over the Ni (5wt%)/C catalyst

Table S2. Summary of yields, purity, and I_G/I_D ratio of CNTs synthesized over different catalysts at 600 ºC.

Sample	CNTs yield(%)	CNTs purity(%)	I_G/I_D
Mo-Ni/C(Mo:Ni=1:9)	26.4	37.4	2
Mo-Ni/C(Mo:Ni=2:8)	30.5	46.3	2.6
Mo-Ni/C(Mo:Ni=3:7)	23.6	32.2	1.7
Ni/C	21.8	25.2	0.8

Table S3. The comparison of the H_2 and CNTs yield of catalysts from the recent literature and this work.

Sample	H_2 yield (%)	CNTs yield (%)	Reference
Mo-Ni/C(Mo:Ni=2:8)	86	30.5	This work
Ni-Mo/MgO	75.1	23.5	J. Environ. Chem. Eng. 2022, 10, 107910
Mo-Ni/C	84	28.3	J. Nanosci. Nanotechnol. 2018, 18, 4387-4392,
NiFeAl	86	26.3	Chem. Eng. Res. Des. 2020, 163,96
CoMo/MgO	30	33	Int. J. Hydrog. Energy 2021, 46, 38175
Ni-loaded MCM-22 c	55	29.8	Int. J. Hydrog. Energy 2011, 36, 13352
